# As3MT and GST Polymorphisms Influencing Arsenic Metabolism in Human Exposure to Drinking Groundwater

**DOI:** 10.3390/ijms21144832

**Published:** 2020-07-08

**Authors:** Farith González-Martínez, Daniel Sánchez-Rodas, Nelson M. Varela, Christopher A. Sandoval, Luis A. Quiñones, Boris Johnson-Restrepo

**Affiliations:** 1 Environmental Chemistry Research Group and Public Health Research Group, University of Cartagena, Cartagena 130015, Colombia; fgonzalezm1@unicartagena.edu.co; 2 Latin American Network for Implementation and Validation of Clinical Pharmacogenomics Guidelines (RELIVAF-CYTED), 28015 Madrid, Spain; nvarela@med.uchile.cl; 3 Center for Research in Sustainable Chemistry, CIQSO, University of Huelva, 21071 Huelva, Spain; rodas@dqcm.uhu.es; 4 Laboratory of Chemical Carcinogenesis and Pharmacogenetics (CQF), Department of Basic-Clinical Oncology (DOBC), Faculty of Medicine, University of Chile, Santiago 8320000, Chile; chris.sandovalp@gmail.com

**Keywords:** arsenic, urinary arsenic metabolites, arsenic speciation, polymorphic variants, arsenic-3-methyl-transferase, glutathione-S-transferase

## Abstract

The urinary arsenic metabolites may vary among individuals and the genetic factors have been reported to explain part of the variation. We assessed the influence of polymorphic variants of Arsenic-3-methyl-transferase and Glutathione-S-transferase on urinary arsenic metabolites. Twenty-two groundwater wells for human consumption from municipalities of Colombia were analyzed for assessed the exposure by lifetime average daily dose (LADD) (µg/kg bw/day). Surveys on 151 participants aged between 18 and 81 years old were applied to collect demographic information and other factors. In addition, genetic polymorphisms (*GSTO2-rs156697*, *GSTP1-rs1695*, *As3MT-rs3740400*, *GSTT1* and *GSTM1*) were evaluated by real time and/or conventional PCR. Arsenic metabolites: As^III^, As^V^, monomethylarsonic acid (MMA), and dimethylarsinic acid (DMA) were measured using HPLC-HG-AFS. The influence of polymorphic variants, LADD and other factors were tested using multivariate analyses. The median of total arsenic concentration in groundwater was of 33.3 μg/L and the median of LADD for the high exposure dose was 0.33 µg/kg bw/day. Univariate analyses among arsenic metabolites and genetic polymorphisms showed MMA concentrations higher in heterozygous and/or homozygous genotypes of *As3MT* compared to the wild-type genotype. Besides, DMA concentrations were lower in heterozygous and/or homozygous genotypes of *GSTP1* compared to the wild-type genotype. Both DMA and MMA concentrations were higher in *GSTM1-null* genotypes compared to the active genotype. Multivariate analyses showed statistically significant association among interactions gene-gene and gene-covariates to modify the MMA and DMA excretion. Interactions between polymorphic variants As3MT*GSTM1 and GSTO2*GSTP1 could be potential modifiers of urinary excretion of arsenic and covariates as age, LADD, and alcohol consumption contribute to largely vary the arsenic individual metabolic capacity in exposed people.

## 1. Introduction

The exposure to inorganic arsenic species (InAs) such as trivalent (As^III^) and pentavalent (As^V^) through drinking groundwater is a global public health issue leading to chronic toxicological effect in humans [1,2,3]. The As^III^ corresponds to arsenite species: AsO_3_^3−^, HAsO_3_^2−^, H_2_AsO_3_^-^ and H_3_AsO_3_ while As^V^ denotes arsenate species: AsO_4_^3−^, HAsO_4_^2−^, H_2_AsO_4_^−^ and H_3_AsO_4_. Inorganic arsenic species are classified by the International Agency for Research on Cancer as Group I type compounds [4]. The World Health Association has recommended a safe level of 10 µg/L as a guideline in drinking water [5]. Reports in several countries of Latin America have shown concentrations of arsenic in groundwater above this risk level [1,6,7,8] and the distribution of arsenic in soils, sediments, vegetables and irrigation water has been recently reviewed in Colombia [9] and Brazil [10]. The presence of arsenic in soils and waters naturally may occur, although anthropogenic activities have been the main contributory factor to the environmental contamination by arsenic, soils naturally enriched by arsenic need more attention.

The metabolism of arsenic in the human body has been proposed through two pathways. The classical pathway suggests a process of sequential reduction and oxidative methylation. InAs is quickly absorbed as arsenite (As^III^) or arsenate (As^V^) in the gastrointestinal tract, where these species are methylated to monomethylarsonic acid (MMA) and dimethylarsinic acid (DMA). MMA and DMA metabolites are less toxic than both inorganic species and more readily excreted in urine [11,12]. The second proposed pathway suggests that arsenic either binds to certain proteins [13] or conjugates with glutathione [14] and after subsequent methylation steps results into two final products: (MMA^V^ and DMA^V^).

The percentages of urinary excretion of arsenic species in humans have been used as biomarkers of the individual metabolic capacity. Therefore, part of the interindividual variation among these species in urine may be due to habits and/or environmental factors such as smoking, alcohol consumption, diet, frequency and duration of arsenic exposure, and demographic, anthropometric factors such as age, sex, body mass index (BMI) and pregnancy [15,16]. In the last decade a series of genetic factors, including genetic polymorphisms in metabolic enzymes have been reported to explain the variation in the arsenic methylation capacity of humans [2,8,17].

**Figure 1:**
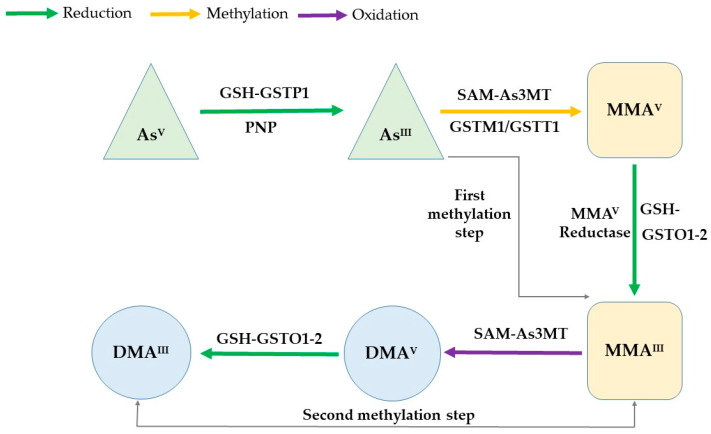
The classical pathway for explain the mechanism of arsenic metabolism. The As^V^ is reducing to As^III^ through a conjugation reaction among Glutathione (GSH as the electron donor) and Glutathione-S-transferase enzymes (GSTP1), as well as purine nucleoside phosphorylase (PNP) enzymes. As^III^ species are methylated to monomethylarsonic acid (MMA^V^) and dimethylarsinic acid (DMA^V^). The enzyme participant is Arsenic (+3)-methyl-transferase (As3MT), using S-adenosyl-methionine (*SAM*-methyl donor). GSH and GSTO1–2 involve in a conjugated reaction to form the intermediate products monomethylarsonous acid (MMA^III^) and dimethylarsinous acid (DMA^III^). The MMA^V^ reductase enzyme is responsible for the reduction of MMA^V^ to MMA^III^.

The main enzymes involved in the arsenic metabolism are classified in three groups: purine nucleoside phosphorylase (PNP), arsenic methyltransferase (AS3MT), and glutathione-S-transferases (GSTs), all of them having genetic polymorphisms [18,19]. PNP enzymes has been proposed as reducing agents of As^V^ [20], expression of As3MT probably plays an important role in the methylation of As^III^ [21,22], and GST are a series of phase II enzymes that detoxify xenobiotics via conjugation reaction with glutathione (GSH). Four members from the GST family including GSTO (isoforms 1 and 2), GSTP1, GSTT1 and GSTM1 could influence the capacities to metabolize arsenic, according the expression level and the presence of different allele variants [2,23,24]. GSTO (ω class) omega 1 reduces pentavalent and methylated arsenic species [25]. Moreover, the GSTO omega 2 reduces only methylated species [26]. Besides, low activity of GSTP1 (π class) enzyme could decrease the detoxification function of GSH [27] and the enzymes GSTT1 (θ class) and GSTM1 (μ class) may facilitate the methylation of inorganic arsenic. Therefore, the absence of their activity could increase the urinary excretion of inorganic arsenic [28] (Figure 1).

Polymorphic variants of *GST* and *As3MT* genes influence the arsenic metabolism giving rise to inter-individual differences. These polymorphic variants are likely to decrease the enzymatic activity, increasing the toxicity of inorganic arsenic, though for *GSTP1* gene the findings were not consistent [29,30]. The *GSTT1-null* and *GSTM1-null* genetic polymorphisms have also been suggested as participants in the arsenic metabolism. Nevertheless, its effects are minors [2,31]. Conversely, *As3MT* and *GSTO* are polymorphisms that have the most evidence associated with the capacities to metabolize arsenic, through the increasing in the expression of the enzymatic activity responsible for the methylation of inorganic arsenic [32,33,34,35]. Genetic polymorphisms in these enzymes have been shown to have differences in frequencies (genotypic and allelic) worldwide, depending on ethnicity/race [36,37].

Previous work performed by our group showed that polymorphisms of *GSTP1*(*rs1695*) increase urinary inorganic arsenic and decrease the primary methylation index in Colombian study population exposed to low and high arsenic concentrations from drinking groundwater [8]. However, the polymorphisms *GSTO* and *As3MT* have not been evaluated, which play an important role in the methylation of inorganic arsenic. There are some knowledge gaps in the arsenic metabolism that have been not elucidated yet. Hence, it is necessary to clarify relationship among metabolites and genotypes.

Therefore, it is necessary to study in the Colombian study population, the polymorphic variant frequencies and their effect on methylation capacity to establish intracellular retention rates of inorganic arsenic. Therefore, the aim of this study was to evaluate the influence of *As3MT* and *GSTs* polymorphic variants on urinary arsenic metabolites and their possible combined effect with the demographic, anthropometric and lifestyles factors.

## 2. Results 

### 2.1. Study Population Characterization

Regarding the demographics, all participants had a mean age of 41.8 ± 13.6 years, being statistically greater in the high exposure group (44.8 ± 14.1) than the low exposure group (40.3 ± 13.3), (*p* = 0.02). The subjects were mostly women (71.5%) and gender was not different between the two groups. BMI was lower in the high exposure group compared to the low exposure group (*p* < 0.01). Results for total arsenic in water was statistically different among the high exposure group (35.5 µg/L) and low exposure group (10 µg/L), (*p* < 0.01). Urinary concentrations of DMA were significantly greater in the high exposure group compared to low exposure group (*p* = 0.03) while total arsenic (µg/g creatinine) was not different between groups. Likewise, the median of the analysis of genotypic frequencies for the polymorphisms evaluated was not different between groups (Table 1).

### 2.2. Arsenic in Drinking Groundwater and Lifetime Average Daily Dose 

Twenty-two groundwater wells from studied municipalities were analyzed for TAs in two different years. The first measurements were in September 2017, with a median of 31.5 µg/L (IQR = 20.6, 39.2). The second measurements were in December 2018, with a median of 33.3 µg/L (IQR = 30.9, 36.7). These values were higher than the guideline reference values of 10 μg/L for arsenic WHO. For the samples collected in 2018, speciation analysis was also performed for As^III^ and As^V^ concentrations with a median of 23.6 μg/L and 9.7 μg/L respectively. In addition, some physicochemical parameters of the groundwater were determined in order to have an overview of drinking water quality (Table S3). The population of study had an overall average of water consumption of 2.5 L/day for an exposure frequency of 365 days/year. The exposure duration was different between the high and low exposure groups (17.9 years and 15.9 years respectively) and the body weight average between both groups was very similar. The median of LADD for the high exposure group to arsenic by drinking groundwater was 0.33 µg/kg bw/day and the HQ 1.1 while the median of LADD for the low exposure group 0.08 µg/kg bw/day and the HQ 0.29 (Table S4).

### 2.3. Genotype Analyses 

Polymorphisms analyzed were consistent with the frequencies expected from the Hardy-Weinberg equilibrium HWE (chi-square test; *p* > 0.05). The analysis of genotypic and allelic frequencies for the polymorphisms evaluated showed the following results: *GSTT1-null* 21.9%, *GSTM1-null* 31.1%, *GSTO2* wild-type 67.5%, heterozygous genotypic variant 27.2% and its homozygous genotypic variant 5.3%, *GSTP1* wild-type 41.7%, heterozygous genotypic variant 41.7% and its homozygous genotypic variant 13.3%, *As3MT* wild-type 41.1%, heterozygous genotypic genotype 41.1% and the homozygous genotypic genotype 17.9%. For the allelic frequencies of *GSTO2-rs156697* Alleles T/C (81/19), *GSTP1*-*rs1695* Alleles A/G (66/34) and *AS3MT*-*rs3740400* Alleles T/G (62/38). In the Table S5 genotypic and allelic frequencies found were compared with other populations studied of Latin American and worldwide. The data showed the variability of each individual independent of their ancestrality.

### 2.4. Total Urinary and Arsenic Species

The Table S6 shows multiple comparisons of urinary arsenic metabolites, corrected by Bonferroni test and adjusted by age and BMI. Changes were observed to MMA (*p* = 0.04) and TuAs (*p* = 0.02) for the differences between high exposure and low exposure groups. Additionally, sample size effect test or Cohen’s d was calculated for each comparison of arsenic metabolite concentrations among the groups (InAs d = 0.21; DMA d = 0.53; MMA d = 0.52; and TuAs d = 0.48).

### 2.5. Univariate Analyses of Polymorphisms and Covariates on Urinary Arsenic Metabolites

Concentrations of urinary arsenic species were compared for genotypes by Wilcoxon rank-sum test and the significance levels were adjusted with the Bonferroni test which showed some statistically significant results as reported in the Table S7. *GSTP1* gene was associated with the urinary excretion of DMA (*p* = 0.04), *As3MT* gene was associated with MMA (*p* = 0.04), and *GSTM1* gene was associated with both DMA (*p* = 0.02) and MMA (*p* = 0.02). The influence of GST and *As3MT* polymorphic variants on urinary concentrations of arsenic metabolites, were evaluated separately by simple Dirichlet regression in a first model in Table 2. The results showed that the *GSTT1*-null variant was associated with InAs, MMA and DMA indicating an increase of InAs (*p* = 0.04) and MMA (*p* = 0.01), and a decrease of DMA (*p* < 0.01). Moreover, the *GSTM1*-null variant showed associated with DMA, indicating an increase (*p* < 0.01). Similarly, the heterozygous/homozygous genotypes of the *GSTO2* and *As3MT* were associated with lower urinary arsenic concentrations of DMA. *GSTP1* and *As3MT* genes had associated with InAs, indicating an increase. The univariate analyses of covariates were significant for MMA with gender (*p* = 0.04) and InAs with alcohol consumption (*p* = 0.03) (Table S8). Results of the univariate analyses are shown in Table 3. 

### 2.6. Multivariate Analyses of Polymorphisms and Covariates on Urinary Arsenic Metabolites

**Figure 2:**
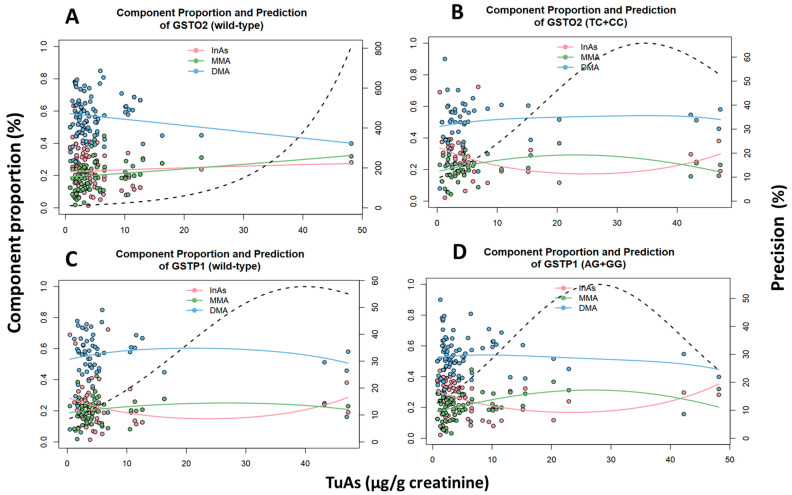
Multivariate Dirichlet regression on the relationship among the urinary arsenic metabolites and total urinary As concentration by genotype adjusted. (**A**) Wild-type genotype of *GSTO2*: InAs (*p* < 0.01), MMA (*p* = 0.01) and DMA (*p* < 0.01). (**B**) Heterozygous+ homozygous genotypes of *GSTO2*: InAs (*p* = 0.27), MMA (*p* < 0.01) and DMA (*p* = 0.01). C. Wild-type genotype of *GSTP1*: InAs (*p* = 0.51), MMA (*p* = 0.02) and DMA (*p* = 0.03). D. Heterozygous + homozygous genotypes of *GSTP1*: InAs (*p* = 0.15), MMA (*p* < 0.01) and DMA (*p* < 0.01).

**Figure 3:**
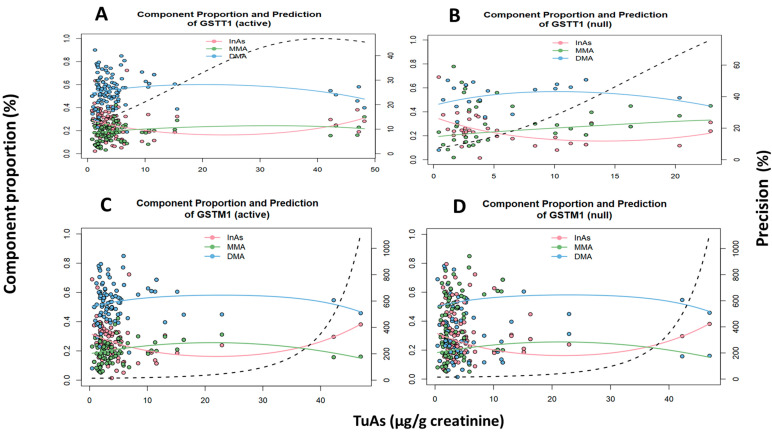
Multivariate Dirichlet regression on the relationship among the urinary arsenic metabolites and urinary As concentration by genotype adjusted. (**A**) Active *GSTT1*: InAs (*p* = 0.70), MMA (*p* = 0.03), DMA (*p* = 0.05). (**B**) Null *GSTT1*: InAs (*p* = 0.71), MMA (*p* = 0.07), DMA (*p* = 0.08) (**C**) Active *GSTM1*: InAs (*p* = 0.32), MMA (*p* = 0.38), DMA (*p* = 0.53) (**D**) Null *GSTM1*: InAs (*p* = 0.06), MMA (*p* < 0.01), DMA (*p* < 0.01).

**Figure 4:**
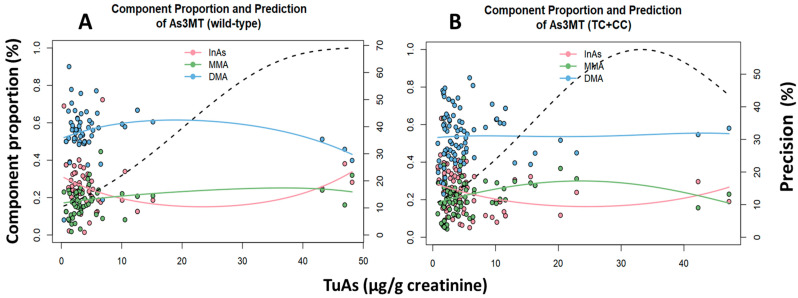
Multivariate Dirichlet regression on the relationship among the urinary arsenic metabolites and urinary As concentration by genotype adjusted. (**A**) Wild-type genotype of *As3MT*; InAs (*p* = 0.18), MMA (*p* < 0.01), DMA (*p* < 0.01). (**B**) Heterozygous+ homozygous genotypes of *As3MT*: InAs (*p* = 0.70), MMA (*p* = 0.06), DMA (*p* = 0.08).

Multivariate Dirichlet regressions of the relationship between urinary arsenic metabolites and genotypes were performed and shown in Figure 2, Figure 3 and Figure 4. Interdependence among the three urinary arsenic metabolites and the total urinary arsenic concentration was adjusted by genotypes, showing the impact of the methylation efficiency in the toxicity of arsenic (Figure 2). For the heterozygous/homozygous variant of *GSTO2*, %DMA was lower compared to the wild-type and tend to increase proportionally with the increase of total urinary arsenic, while that %MMA trend to decrease. For the heterozygous/homozygous variant of *GSTP1*, %DMA was lower compared to the wild-type and decreased proportionally with the increase in total urinary arsenic. Similarly, %MMA tend to decrease their concentration with the increase of total urinary arsenic concentration.

For *GSTT1* gene, the Figure 3 showed for the active variant a decreasing trend for %DMA, as well as a total urinary arsenic concentration increase, while %MMA tend to decrease. Conversely, the *null* variant had no significance. For *GSTM1* gene, the relationship among the active variant and the metabolites and the total urinary arsenic concentration had no significance (Figure 3C), while in the *null* variant carrying people, %DMA and %MMA were higher and decreased proportionally with the increase in total urinary arsenic (Figure 3D). In relation to the *As3MT* gene, the wild-type genotype showed a decreasing trend for urinary %DMA and %MMA, as well as total urinary arsenic increase. The heterozygous/homozygous genotypes had no significant association either with total urinary arsenic concentration or metabolites (Figure 4B).

The results from the multivariate logistic regression analyses are presented in Table 4. The analysis of the influence of polymorphic variants and potential confounders on InAs, MMA and DMA, adjusted by interaction term showed three statistically significant final models. In the final model for %MMA (cat. 10–20% = normal, > 20% = high) the result was statistically significant (R^2^ = 0.40; likelihood-ratio test, *p* < 0.01). The gene-gene interactions (*As3MT-GSTM1*), gene-demographic factors (*As3MT*-Age) and gene-habits (*As3MT*-Alcohol consumption), generated larger β coefficients and a moderate determination coefficient for %MMA. In the final model for %DMA (cat. 60–80% = normal, < 60% = low) the result was statistically significant (R^2^ = 0.20; likelihood-ratio test, *p* < 0.01). The gene-gene interactions (*GSTO2-GSTP1*), and gene-environmental factors (*GSTO2*-LADD and *GSTP1*-LADD), generating lower β coefficients and a determination not large coefficient. On the other hand, the effect of interactions term in the final model for %InAs was not relevant and had poor statistical strength was obtained.

## 3. Discussion

This study gives additional support regarding the role of single nucleotide polymorphisms (SNPs) on arsenic metabolism and body burden. To interpret the findings of the polymorphisms associated with urinary arsenic metabolites, in the present study the interactions between the three arsenic species (%InAs, %MMA, and %DMA), allowed to distinguish between higher or lower %DMA to the other two species, which could have a biological to the interpretation of “fast secondary methylation step”. Similarly, we evaluated higher or lower %InAs in relation to %MMA and %DMA. This analysis could have a biological interpretation of “fast primary methylation step”. Several authors have proposed the use of excretion ratios of MMA/InAs and DMA/MMA in the urine, also referred as primary methylation index (PMI) and secondary methylated index (SMI), respectively and used as surrogate biomarkers of the arsenic metabolic capacity [18,38,39,40]. Nevertheless, SMI and PMI measurements only use information about two of the three species and completely fail to account for the dependence between the arsenic species outcomes which does not have a clear biological interpretation [41,42]. In the present study we fitted a Dirichlet regression on genotype adjusted for genome wide markers, which might be a good way to describe changes in the urinary arsenic metabolites.

Our findings indicated that for people carrying heterozygous/homozygous genotypes of *As3MT* (*rs3740400*), the %MMA was higher compared to the wild-type genotype. These results are similar to reported in European [18,43], Asian [15,44] and Latin American populations [39,45]. For *GSTP1*(*rs1695*), the %DMA was lower compared to the wild-type genotype. These finding are consistent with the data found in Chilean individuals [31] and contrary to reports in Vietnamese and North American populations [30,46]. Likewise, the deletions such as *GSTM1*, the %DMA, %MMA and %InAs were higher in the group with null genotype compared to the active genotype. In Chilean population similar data were found for %MMA and %InAs, but no for DMA [31]. In this population there is not clear association between *GSTM1* gene and the arsenic metabolite excretion levels was found [2]. The focus of these differences may be the direct or indirect relationship that these genes have with the degree of methylation of urinary arsenic species. One of the current hypothesis of the arsenic methylation efficiency in the toxicity of arsenic supposes that a lower excretion of DMA is the result of a decreasing in the degree of methylation of the inorganic forms, generating a minor individual metabolic capacity of arsenic [34,47]. Methylated species of arsenic are recognized to be less toxic than inorganic ones, the methylation has been considered to be a detoxification mechanism for arsenic in the mammals [12]. Although reports in Chilean populations are opposed to the plausibility of this hypothesis, these inconsistent findings need major evidence [48]. Therefore, our results are probably plausible from this first hypothesis, because As3MT and GSTO2 enzymes are related directly with the methylation of inorganic arsenic species, both oxidative and reductive metabolic pathways of As^III^. *As3MT* (*rs3740400*) genotype is recognized for its importance in the enzymatic expression of As3MT to methylate As^III^ in species MMA^III^ and DMA^III^ [6,49] and the enzymes of *GSTO2* *(rs156697)* genotype could reduce the methylated species MMA^V^ and DMA^V^ [26]. Although in our study *GSTO2* polymorphism (*rs156697*) did not show statistical significance with any of the urinary arsenic metabolites. In the present study, DMA was the major arsenic metabolite excreted after exposure to arsenic via drinking water, followed by InAs, and MMA. These metabolites have been typically reported in the literature as excretion ratios in ranges of 60–80%, 10–30%, 10–20% for DMA, MMA and InAs, respectively [50,51,52]. However, these may be variable between individuals and populations, particularly DMA, considered the dominant methylated arsenic species in urine samples [53,54]. Although, several authors also have reported different values for DMA [41], currently there is no consensus on the urinary excretion percentages to be considered as guiding parameters of arsenic toxicity at the individual level. Similar conclusions have been estimated for MMA [55,56].

Besides, GSTP1, GSTT1 and GSTM1 enzymes participate in detoxification of arsenic but not directly methylating the inorganic arsenic [28]. *GSTP1* heterozygous/homozygous variants showed a lower concentration of urinary of InAs compared to the wild-type genotype. Heterozygous genotype of *GSTP1* (AG) could have a lower reduction capacity from As^V^ to As^III^, contributing to MMA^V^ metabolism [30]. As consequence of low activity of the GST variants, it is possible decrease the detoxification function of GSH, altering the excretion of urinary arsenic metabolites [27]. Besides, GSTM1 may facilitate the methylation of inorganic arsenic. Thus, people with null variants of *GSTM1* could not efficiently methylated As^III^ giving rise to an increase in the amount of urinary inorganic arsenic, and also to an increase in the %MMA. It has been suggested that the increased excretion of MMA indicates that the second methylation step is inhibited by two hypotheses: excess of inorganic arsenic or the saturation of the enzymatic conversion of MMA to DMA [56]. Interestingly, the relation among the urinary arsenic metabolites and the total urinary arsenic concentration in the present study was evidenced to %DMA with a trend to increase proportionally with the increase of total urinary arsenic. Moreover, MMA presented a trend to decrease proportionally with the increase of total urinary arsenic. In the first case, there could be an influence of *GSTO2* and *As3MT* homozygous/heterozygous genotypes and in the second case by *GSTP1* homozygous/heterozygous genotypes and *GSTT1* and *GSTM1*-*null* variants. Regarding to the prevalence of GST polymorphisms, *GSTT1* and *GSTM1* null variants were similar to those reported for other Latin-American studies [2,31]. *GSTP1* (*rs1695*9) heterozygous and homozygous genotypes have been also reported with a high prevalence in populations from different countries [46,57]. Likewise, *GSTO2*(*rs156697*) polymorphic variants have been evaluated in some US [46] and Poland [18] studies and the prevalence are higher. Furthermore, *As3MT* (*rs3740400*) heterozygous and homozygous genotypes have been described with similar prevalence to those reported in the present study [22].

The effects of toxicity of inorganic arsenic may be accelerated with an increase on arsenic exposure dosage [58]. The limited methylation capacity associated with an increased dosage by excessive InAs could be explained through the inhibition of the second methylation step [59,60]. In our study, the concentration of arsenic methylated species followed the trend to increase in subjects exposed to major dosage. There is no evidence suggesting a saturation in the arsenic metabolic capacity, therefore, other explanatory mechanisms including genetic and environmental factors may be possible [18]. Lifetime average daily dose have been reported as an important human risk tool for exposure assessment to many pollutants [61]. In the present study, lifetime average daily dose in exposed subjects to arsenic was slightly higher to the reference dose (0.3 µg/kg bw/day). This may explain the risk generated by exposure to water consumption with high concentrations of arsenic, which could show adverse health effects after >20 years continuous exposure (cardiovascular disease, hypertension, neurological diseases, respiratory diseases, atherosclerosis, cardiometabolic diseases and several kind of cancers) [62,63,64,65,66,67].

To explain the influence of these polymorphisms and covariates on urinary arsenic metabolites, the multivariate analyses to %MMA showed significant associations between *As3MT*, *GSTM1*, age, BMI and alcohol consumption. The final models were adjusted by interactions (gene-gene, gene-physical factors and gene-habits) explaining 40% of the variation (R^2^ = 0.40; *p* < 0.001). As *As3MT* and *GSTM1* polymorphic variants have effects on MMA in the same pathway, their interaction may increase the strength of the association in the final model. Moreover, in the scientific literature have been reported interactions among *GSTP1* variant and *GSTM1*-*null*, resulted in significantly reduced GST enzymatic activity [28], which could affect arsenic metabolism [29,46]. Nevertheless, for *As3MT* and *GSTM1* no findings were obtained, therefore more consistent evidence is needed. Covariates tested as potential confounders, their interactions were analyzed including *As3MT*-age and *As3MT*-alcohol consumption to explain changes in MMA urinary excretion. Recent reports have suggested that age of subjects is related with methylation capacity [48]. Adults ≥ 63 may have significantly lower urinary excretions of DMA but higher those of InAs compared with young subjects [68]. Due to these contradictory findings and that aging can probably be associated with a variety of functional changes in the organs involved in the metabolism or retention of the metabolites of arsenic, their effect on arsenic metabolism needs to be confirmed [38]. On the other hand, in this work the alcohol consumption showed significant association with MMA and its interaction with *As3MT* enhance its effects. In relation to that, some authors suggest as a potential mechanism that the presence of GST polymorphic genes could alter the capacity to conjugate lipid peroxidation products, cytotoxic compounds, and free radicals generated during alcohol metabolism is reduced [69,70]. Other authors observed a relationship among *As3MT* alleles, high arsenic level and the presence of glutathione (GSH) in the reaction medium. These authors suggested that individual variations in the GSH metabolism should also be included among the modifiers of arsenic metabolism in humans [25,30,71].

Limitations of this study are related to the non-measurement of other potential routes of arsenic exposure such as agriculture, mining and landfills, which can be sources of occupational arsenic exposure. Likewise, although the forms of organic arsenic were not measured in our study, when we compared the total urinary arsenic concentration with the total sum of urinary arsenic metabolites, the median was very similar. These small differences could be due to other organic arsenic species, such as arsenobetaine (AsB) (abundant in crustacean seafood) increasing the total urinary arsenic concentration and excreted unchanged into the urine [38,72]. Therefore, this organic arsenic form appears to not influence DMA concentration. Whereby the urinary metabolites found in this study are not related to seafood consumption. Besides, other covariates were evaluated through surveys with which we could not exclude the possibility of an information bias. Nevertheless, strengths of the study included assessing the influence of genetic polymorphisms and their interactions to arsenic metabolism will provide abundant epidemiological data to health institutions. This will allow the understanding of the individual and population differences to identify susceptible groups in Colombia, making better risk estimates of adverse effects to arsenic from drinking groundwater intake. With this information, a readjustment of strategic planning in public health could be achieved in the case of contamination of drinking water in high-risk populations. Nonetheless, to obtain greater power and statistical strength with estimates and predictions about arsenic metabolism, it is necessary to evaluate the interactions among different isoforms of other genes associated with potential factors involved in the process, which could improve the usefulness of the results obtained.

## 4. Materials and Methods

### 4.1. Subject of Study and Sample

This study was conducted from September of 2017 to December of 2018 and included 151 adults aged between 18 and 81 years old selected from the municipalities of Margarita and San Fernando, in Bolivar department, Colombia. Margarita (9°09′11″ N 74°17′17″ W) is located at an altitude of 20 m asl with average of temperature of 28 °C, extension of 295 Km^2^ and a population of 9876 inhabitants, about 90% of them living in the rural areas. San Fernando (9°12′43″ N 74°19′23″ W) is located at an altitude of 33 m asl with average of temperature of 27.8 °C, extension of 288 Km^2^ and a population of 13,753 inhabitants, about of 80% of them living in the rural areas (Figure S1). Eligible participants enrolled in this study have been living in these municipalities for at least 5 years and consume groundwater on a regular basis. Exclusion criteria were as follows: occupational exposure to arsenic, medical history of renal or hepatic disease, exposure to drug therapy within the two months before sampling, consumption of seafood within 48 h before sampling [53], and a family relationship to another study subject. This study was approved by the Human Research Ethical Committee of the University of Cartagena, Colombia (25 May 2017) and was registered on the University of Cartagena Register (#403962017, 2 June 2017).

The sample size was determined based on a type I error of 5% and power of 80%. Based on previous data, using Stata^®^ software (V12, Stata Corp. LP, College Station, TX, USA); the lowest expected frequency for heterozygous and/or homozygous variants (*GSTP1*) was used [8]. Besides, it was included the lowest expected frequency of all the urinary arsenic species percentages (MMA = 18.3%) [8], resulted in a target sample size of 140 subjects. To account for possible damage to blood specimens for DNA testing or incomplete processing of the questionnaire, the sample size was increasing 10% (14 subjects). Thus, a total of 154 donors were recruited, 3 subjects were excluded because they did not fulfill the parameters previously cited. Therefore, the final population consisted of 151 participants.

A survey was used to assess the demographic and anthropometric information about study participants, including lifestyle and smoking history. Information regarding the smoking habits and alcohol consumption was obtained through a self-administered questionnaire, with a supplementary interview. Subjects were asked whether they were lifelong nonsmokers or current smokers. We use the Brinkman’s index of tobacco addiction [73] to generate two groups related to smoking: nonsmoker and smoker group. Similarly, to evaluate alcohol consumption, we used a cut-off value at least 280 g per week [74].

### 4.2. Sample Collections

#### 4.2.1. Groundwater Collection

Twenty-two randomly selected groundwater wells sites were selected in the Margarita and San Fernando municipalities (Figure S1). Water samples were collected in 250 mL polyethylene bottles, previously treated with 10% HNO_3_ for 2 h, rinsed with ultra-pure water (18 MΩ·cm; distilled and deionized water) and dried (to avoid heavy metal contamination). Containers were also rinsed, 3 times with the same sampling water, before taking. After sample collection, they were kept under refrigeration, transported, and stored at the laboratory, until analyzed.

#### 4.2.2. Urine Collection

Urine samples from study participants were collected in 25 mL polyethylene bottles, previously treated with 10% HNO_3_ for 2 h, and rinsed with ultra-pure water. The urine collection of each subject was a spot sample in the morning as the first one. To reduce variation of the arsenic concentrations among individuals, those were adjusted by creatinine. Samples were kept under refrigeration, transported at the laboratory and then lyophilized immediately (FreeZon 4.5 Liter Benchtop Freeze Dry System) to avoid possible oxidation of inorganic arsenic species. The samples were reconstituted with 1.8 mL ultra-pure water in Eppendorf tubes and filtered using PTFE syringe filters (0.45 µm) (Whatman, GE Healthcare Life Sciences, UK) which analyzed immediately.

#### 4.2.3. Blood Samples Collection

Peripheral blood samples from study participants were collected by venous puncture using lead-free vacutainer tubes containing EDTA as an anticoagulant (Becton Dickinson, Inc., Rutherford, NJ, USA). These were collected from all the participants at the time of enrollment and were transported at the laboratory until analysis. Genomic DNA was extracted through the High Pure PCR Template Preparation Kit (catalog number 11796828001; Roche Diagnostics GmbH, Mannheim, Germany).

### 4.3. Groundwater Quality and Arsenic Determination

Various physicochemical water quality parameters were measured with the appropriate instruments. These parameters included turbidity, pH, conductivity, temperature, dissolved oxygen, chloride, fluoride, nitrite, nitrate, magnesium and others. Sample measurements were determined in situ using a turbidimeter HI83414 (Hanna instruments) for Turbidity (NTU) and a portable pH/ISE/Conductivity/RDO/DO/Temperature Multiparameter Meter (Thermo Scientific Orion Star A329) for conductivity (Ω/cm), pH, temperature (°C), and Dissolved Oxygen (mg/L). Chloride (mg/L) and fluoride (mg/L) were measured in the laboratory using a benchtop multiparameter pH/ISE (Thermo Scientific Orion 4-Start Plus) with the respective ion selective electrodes. Nitrite (mg/L), nitrate (mg/L) were measured by colorimetry using a spectrophotometer Genesys 10S UV-VIS (Thermo Scientific). Total dissolved solid (mg/L), alkalinity (mg/L), total hardness (mg), calcium (mg/L), and magnesium (mg/L) were determined followed the standard method, American Public Health Association (APHA), American Water Works Association (AWWA) and World Economic Forum (WEF). Total arsenic (TAs) concentrations in groundwater samples were digested with HNO_3_ and HClO_4_. Then it was measured by Hydride Generation-Atomic Absorption Spectrometer (HG-AAS) equipped with a VP-100 continuous flow vapor generator system (Thermo Scientific iCE 3500). The speciation analysis for inorganic arsenic species (arsenite and arsenate) in duplicated groundwater samples was performed by a High-Performance Liquid Chromatography (HPLC) coupled to Hydride Generation-Atomic Fluorescence Spectrometry (HG-AFS); PSA 10.055 Millennium Excalibur system (PS Analytical Ltd., Orpington, Kent, UK).

### 4.4. Exposure/Risk Assessment

Human exposure to arsenic in groundwater was calculated using the lifetime average daily dose (LADD) which is the amount of daily arsenic exposed from one or more sources and is expressed on the basis of µg of arsenic per kg body weight per day (μg/kg bw/day), according the following equation:

LADD = (C × IR × ED × EF)/(BW ×AT)(1)

where C is arsenic concentration in water (µg/L), IR is the water intake rate per day (L/day), ED is exposure duration (years), EF is the exposure frequency (days/year), BW is the body weight (Kg), and AT is the average time (day). From there the hazard quotient (HQ) was determined as follows:

HQ = LADD/RfD(2)

The RfD (reference dose) for arsenic is 0.3 µg/kg bw/day from U.S. Environmental Protection Agency (US EPA), for non-cancer outcome such as hyperpigmentation, keratosis and possible vascular complications [75]. Results of HQ above 1, indicate an increased risk of adverse health effects.

### 4.5. Instrumental Analysis for Urinary Arsenic Speciation

Urinary arsenic species (As^III^, As^V^, MMA and DMA) were determined using HPLC-HG-AFS [76,77]. Urine samples were eluted using a Hamilton PRP-X100 anion-exchange column of 250 mm × 4.1 i.d., particle size 10 µm (Hamilton Company, Reno, NV, USA) with KH_2_PO_4_/K_2_HPO_4_ aqueous buffer as mobile phase (pH 5.8), a flow rate of 1.0 mL/min. For total urinary arsenic (TuAs) determination, samples were digested with HNO_3_ and HClO_4_ to convert the arsenic species to inorganic ones and then these were measured by HG-AFS.

### 4.6. Quality Assurance/Quality Control.

We used the blank procedure to determine the limit of detection (LOD) and limit of quantification (LOQ) of the method for total arsenic in water obtaining values of 0.7 µg/L and 1.2 µg/L, respectively. Six-point standard calibration curves were used to quantify the sample concentrations from peak areas and the regression coefficients were above of 0.995. Urinary arsenic species concentrations were analyzed as As^III^, As^V^, MMA and DMA. Also, InAs was the sum of inorganic species (As^III^ + As^V^). Seronorm™ Trace Elements Urine was used as an accuracy control for the analyses (reported 106 ± 2 µg/L of total As) (Seronorm Pharmaca, Billingstad, Norway) to check the recovery of arsenic species by HPLC-HG-AFS, obtaining a sum of 107.8 ± 2.4 µg/L, which corresponded to 99.5 ± 2.1 µg/L of As^V^ and 8.3 ± 0.3 µg/L of DMA. Limits of detection (LOD) for urinary arsenic species were as the follow: As^III^ = 0.17, As^V^ = 0.38, MMA = 0.30 and DMA = 0.45 µg/L. Urinary arsenic concentrations were adjusted by dilution to eliminate variation from fluid balance. Therefore, urinary creatinine was determined by spectrophotometer Genesys 10S UV-VIS (Thermo Scientific, USA) and urinary total arsenic concentrations were expressed as µg As per g creatinine.

### 4.7. Genotyping Polymorphisms

Genomic DNA was isolated from peripheral blood samples through the High Pure PCR Template Preparation Kit (catalog number 11796828001; Roche Diagnostics GmbH, Mannheim, Germany). DNA samples were quantified at 260/280 nm using a nanodrop spectrophotometer (model DS-11, FX Series Spectrophotometer, USA). Deletions (*GSTT1-null,* and *GSTM1-null*) were determined by direct PCR and total absence or presence of GSTT1*0 and GSTM1*0 gene was determined in a G-Storm Thermocycler (model GS00482, IDT Fermelo-Biotec, Chile). Beta Globin and Cytochrome P4501A1 were used as an internal positive amplification control for *GSTT1* and *GSTM1*, respectively [78]. Heterozygous and homozygous non-null individuals could not be differentiated; therefore, null genotypes reported elsewhere are actually double null genotypes. The charging procedure of the amplification products was used by electrophoresis (80 V, 2 h) on a 4% agarose gel (Bio-Rad Lab., Richmond, CA, USA) and detected by Syber Safe DNA (Invitrogen^®^), staining under an ultraviolet illuminator, and photographed. The substitutions (*GSTP1-rs1695*; Assay ID:C_3237198_20, *GSTO2-rs156697*; Assay ID:C_3223136_1 and *As3MT-rs3740400*; Assay ID:C_27510174_10) were evaluated by Real-Time PCR (RT-PCR), using *TaqMan*^®^ Genotyping Assays (Thermo Fishier Scientific Inc.^®^; C. Number: 4351379 and 4362691) in a Stratagene^®^ Mx3000P Real-Time PCR system (Agilent Technologies, Waldbronn, Alemania). The context sequences for *TaqMan^®^* probes and primers for PCR are listed in Table S1 and the PCR conditions for the five genes are detailed in Table S2. The reference sequence of each gene was based on GenBank sequences at NCBI (https://www.ncbi.nlm.nih.gov/genbank). For quality assurance purposes, we randomly choose a 20% of the samples for (a) repetition of the analysis and (b) PCR and Restriction fragment length polymorphism (RFLP) analysis for coincidence for RT-PCR analyzed samples or c) confirmation by sequencing (ABI Prism 3100 Genetic Analyzer), when the PCR and RFLP and RT-PCR analyses were not coincident.

### 4.8. Statistical Analyses 

Kolmogorov–Smirnov’s one sample test was used to check for normal distribution of data for each variable. Because concentrations of arsenic in groundwater and urinary arsenic species did not have a normal distribution, hence the estimation of their medians and interquartile ranges were used to statistical description. Deviation of Hardy-Weinberg equilibrium (HWE) was used for a quality control measurement, *GSTP1-*rs1695, *GSTO2-*rs156697 and *As3MT-* rs3740400 genotype distributions were tested using the chi-square χ^2^-test (*p*> 0.05). Genotype frequencies were calculated as the proportion of individuals with a given genotype by dividing into the total number of participants and the allelic frequencies were calculated as the frequencies of heterozygous and homozygous alleles by dividing into the total allelic varieties. The study population was divided into two subgroups for purpose of analysis: participants with low-exposure doses (LADD ≤ 0.3 µg/kg bw/day) made up one group and those with high-exposure doses (LADD > 0.3 µg/kg bw/day) forming the second group. Pairwise comparisons were performed between low and high exposure dose, using parametric and nonparametric tests (Wilcoxon rank sum test, χ^2^-test, *t*-test). For multiple comparisons by genotypes Bonferroni correction was used. A complementary analysis to understand the magnitude. To understand the significance differences between low and high exposure, effect size test was performed [79]. To analyze the polymorphism influence on urinary arsenic excretion metabolites, Dirichlet regression and Logistic regression multivariate were used. The urinary arsenic species were incorporated in the analysis as dependent variables while the polymorphisms and LADD were incorporated as independent variables. Interaction terms among the main genotypes that provided a *p*-value < 0.2 through of genetic dominant models (wild-type vs heterozygous + homozygous genotype) and potential confounders (age, BMI, smoking history and lifestyle) were taken into account. Dominant models over additive model are considered on study population with a better biological sense. The urinary arsenic species were incorporated in the analysis as dependent variables, the polymorphisms and LADD as independent variables. The multicollinearity of independent variables was assessed calculating of variance inflation factor (VIF). The statistical analyses and geospatial data (study site map in the Figure S1) were performed using R software environment [80]. 

## 5. Conclusions

The gene-gene interactions between polymorphisms As3MT*GSTM1 and GSTO2*GSTP1 could be potential modifiers of urinary arsenic metabolites, increasing MMA and decreasing DMA. Additionally, synergistic effect among these polymorphisms and age, LADD of arsenic and alcohol consumption might vary the arsenic individual metabolic capacity a large part. This is an important issue to consider, particularly in this Colombian study population from where the inhabitants are exposed to arsenic in from groundwater water supply.

## Figures and Tables

**Table 1:** General characteristics of the study population.

Characteristics	High Exposure	Low Exposure	Total	*p*-Value
No. Subjects, n (%)	50 (33.1)	101 (66.9)	151 (100)	
Male, n (% Col.) ^a^	15 (30)	28 (27.7)	43 (28.5)	0.77 *
Female, n (% Col.)	35 (70)	73 (72.3)	108 (71.5)	
Age years, mean ± SD ^b^	44.8 ± 14.1	40.3 ± 13.3	41.8 ± 13.6	0.02¤
BMI ^c^, kg/m^2^, mean ± SD	22.1 ± 3.5	26 ± 4.1	24.7 ± 4.3	<0.001¤
Water total arsenic, µg/L, median (IQR) ^d^	35.5 (31.7, 37.3)	10 (10,10)	33.3 (30.9,36.7)	<0.001 †
No. of wells in each place, mean	22	1	23	
Residence years, median (IQR)	17.9 (14.2,20.6)	15.9 (14.2,20.6)	16.9	0.32 †
Urinary TuAs ^e^, µg/g creatinine, median (IQR)	4.2 (3.1,7.8)	3.6 (2.7,7.0)	4.0 (2.7,7.0)	0.07 †
Urinary InAs ^f^, µg/L, median (IQR)	0.80 (0.25,1.33)	0.80 (0.50,1.25)	0.80 (0.5,1.3)	0.97 †
Urinary MMA ^g^, µg/L, median (IQR)	0.80 (0.40,1.5)	0.60 (0.30,1.0)	0.60 (0.30,1.3)	0.08 †
Urinary DMA ^h^, µg/L, median (IQR)	2.1 (1.4,3.1)	1.5 (1.1,2.7)	1.7 (1.1,2.8)	0.03 †
Cigarette smoking, n (% Col.)				
Smoker	3 (6.0)	12 (11.9)	15 (9.9)	0.25 *
Non-smoker	47 (94)	89 (88.1)	136 (90.1)	
Shellfish consumption, n (% Col.)				
≥2 per week	4 (8.0)	12 (11.9)	16 (10.6)	0.46 *
<2 per week	46 (92)	89 (88.1)	135 (89.4)	
Water consumption, n (% Col.)				
≥2 L per day	46 (92)	94 (93)	140 (92.7)	0.70 *
<2 L per day	4 (8.0)	7 (6.9)	11 (7.3)	
Alcohol consumption, n (% Col.)				
≥5 glass weekend	8 (16.0)	29 (28.7)	114 (75.5)	0.08 *
None	42 (84)	72 (71.3)	37 (24.5)	
Genotypes				
GSTT1, n (% Col.)				
Null	11 (22)	22 (21.8)	33 (21.9)	0.97 *
Active	39 (78)	79 (78.2)	118 (78.2)	
GSTM1, n (% Col.)				
Null	18 (36)	29 (28.7)	47 (31.1)	0.36 *
Active	32 (64)	72 (71.3)	104 (68.9)	
GSTP1-rs1695, n (% Col.)				
AG + GG	26 (52)	62 (61.4)	88 (58.3)	0.27 *
AA (wild-type)	24 (48)	39 (38.6)	63 (41.7)	
GSTO2-rs156697, n (% Col.)				
TC + CC	18 (36)	31 (30.7)	49 (32.5)	0.51 *
TT (wild-type)	32 (64)	70 (69.3)	102 (67.5)	
AS3MT-rs3740400, n (% Col.)				
TG + GG	29 (58)	60 (59.4)	89 (58.9)	0.86 *
TT (wild-type)	21 (42)	41 (40.6)	62 (41.1)	

Note: * χ^2^-test. ¤ *t*-test. † Wilcoxon rank sum test. HWE (χ^2^-test; p > 0.05). ^a^ % Col. = proportion of individuals with characteristics by dividing into the total number of in each column. ^b^ SD = Standard deviation. ^c^ BMI = Body mass index. ^d^ IQR = Interquartile range defined as the range from the 25th percentile to the 75th percentile. ^e^ TuAs = total urinary arsenic. ^f^ InAs = inorganic arsenic (As^III^ and As^V^). ^g^ MMA = Monomethylarsonic acid. ^h^ DMA = Dimethylarsinic acid.

**Table 2:** Univariate analyses of polymorphic variants *GST* and *As3MT* on urinary arsenic metabolites.

		Urinary Arsenic Species								
Genotypes		InAs ^b^			MMA ^c^			DMA ^d^		
	*n*	β	SE ^a^	*p*-Value	β	SE	*p*-Value	β	SE	*p*-Value
GSTT1										
Null	33	0.34	0.29	0.04 *	0.45	0.17	0.01 *	0.47	0.16	0.004 *
Active	118	ref.			ref.			ref.		
GSTM1										
Null	47	0.24	0.16	0.13	0.17	0.16	0.27	0.39	0.15	0.009 *
Active	104	ref.			ref.			ref.		
GSTP1 (rs1695)										
AG + GG	83	0.46	0.15	0.002 *	0.08	0.15	0.58	0.02	0.14	0.86
AA (wild-type)	68	ref.			ref.			ref.		
GSTO2 (rs156697)										
TC + CC	49	0.03	0.15	0.84	−0.07	0.16	0.61	−0.47	0.15	0.001 *
TT (wild-type)	102	ref.			ref.			ref.		
As3MT(rs374040)										
TG + GG	89	−0.58	0.15	<0.001 *	−0.24	0.15	0.11	−0.63	0.14	<0.001 *
TT (wild-type)	62	ref.			ref.			ref.		

Note: β = regression coefficient of genetic dominant model compared to the wild-type on urinary arsenic species in genotypic substitutions and genetic basic model *null* compared to active in genotypic deletions. * Statistical significance univariate analysis through Dirichlet regression (*p* < 0.05); ^a^ SE = Standard error; ^b^ InAs= inorganic arsenic (As^III^ and As^V^); ^c^ MMA =monomethylarsonic acid; ^d^ DMA = dimethylarsinic acid.

**Table 3:** Univariate analyses of demographic characteristics and lifestyle on urinary arsenic metabolites.

	Urinary Arsenic Species									
Covariates		InAs ^b^			MMA ^c^			DMA ^d^		
	*n*	β	SE ^a^	*p*-Value	ß	SE	*p*-Value	β	SE	*p*-Value
TAs in water (µg/L), median (range)	26.1 (10,39.4)	−0.09	0.77	0.24	−0.03	0.52	0.55	0.12	0.09	0.18
LADD (µg/kg-bw/d), median (range)	0.23 (0.09,0.34)	−10.3	7.9	0.19	0.64	7.5	0.93	9.6	9.5	0.31
Urinary TuAs ^e^ (µg/g creat.), median (range)	4.0 (2.7,7.0)	−0.03	12.5	0.84	0.18	0.08	0.03*	−0.15	0.15	0.29
Age (years), mean ± SD ^f^	41.8 ± 13.6	−0.12	0.08	0.10	−0.05	0.05	0.32	0.18	0.09	0.04*
BMI ^g^, kg/m^2^, mean ± SD	24.7 ± 4.3	−11	0.24	0.64	−0.14	16.4	0.39	0.25	0.28	0.37
Sex										
Female	43	−1.44	2.3	0.53	−0.98	1.6	0.53	2.41	2.7	0.37
Male	108	ref.			ref.			ref.		
Smoking Habit										
Yes	15	−1.56	3.5	0.65	−2.92	2.4	0.22	4.5	4.1	0.27
No	136	ref.			ref.			ref.		
Alcohol consumption										
≥5 glass per week	37	−0.23	2.4	0.92	−1.73	1.6	0.29	1.95	2.8	0.49
No consumption	114	ref.			ref.			ref.		
Water consumption										
≥2 L per day	140	1.06	3.9	0.79	0.64	2.7	0.81	−1.72	4.7	0.72
<2 L per day	11	ref.			ref.			ref.		
Shellfish and/or fish										
≥2x per week	16	−4.2	3.6	0.24	1.19	2.4	0.63	3.03	4.2	0.47
<2x per week	135	ref.			ref.			ref.		

Note: β = regression coefficient of related covariates categories compared to unrelated covariates categories on urinary arsenic species. * Statistical significance univariate analysis through Dirichlet regression (p < 0.05); ^a^ SE = Standard error. ^b^ InAs =Inorganic arsenic (As^III^ and As^V^). ^c^ MMA = Monomethylarsonic acid. ^d^ DMA = Dimethylarsinic acid. ^e^ TuAs= total urinary arsenic by creatinine adjusted. ^f^ SD = Standard deviation. ^g^ BMI = Body mass index.

**Table 4:** Multivariate analyses of polymorphic variants on relative urinary arsenic metabolites, adjusted by covariates.

Urinary Arsenic Species	β	SE ^a^	*p* Value	*R* ^2^	*p* > chi ^2^
MMA ^b^ (cat. 0 = normal, 1 = high)				0.40	<0.001 *
As3MT ^c^ (TG + GG vs. TT)	15.8	5.9	0.007 *		
GSTM1 ^d^ (Active vs. null)	3.4	0.90	0.01 *		
Age ^e^ (cat. 0 = 18–59, 1 = >60)	0.86	0.69	0.21		
BMI ^f^ (cat. 0 = low, 1 = high)	−1.2	0.39	0.04 *		
Alcohol consumption (0 = no, 1 = yes)	1.6	1.3	0.22		
LADD ^g^ (cat. 0 = low, 1 = high)	2.9	0.9	0.001 *		
As3MT-GSTM1 (interaction 1)	2.3	0.95	0.01 *		
As3MT-Age (interaction 2)	−3.9	1.4	0.006 *		
As3MT-BMI (interaction 3)	−1.9	1.6	0.23		
As3MT-Alcohol (interaction 4)	4.7	2.0	0.01 *		
DMA ^h^ (cat. 0 = normal, 1 = low)				0.20	<0.001 *
GSTO2 ^i^ (TC + CC vs TT)	2.7	1.03	0.009 *		
GSTP1 ^j^ (AG + GG vs. AA)	1.1	0.53	0.05		
GSTM1 ^d^ (Active vs. null)	−1.07	0.49	0.03 *		
Age ^e^ (cat. 0 = 18–59, 1 = >60)	−1.39	0.73	0.05		
LADD ^g^ (cat. 0 = low, 1 = high)	−0.56	0.62	0.36		
GSTO2-GSTP1 (interaction 1)	−2.3	1.06	0.03 *		
GSTO2-GSTM1 (interaction 2)	1.92	1.08	0.07		
GSTO2-LADD (interaction 3)	−2.1	1.02	0.04 *		
GSTP1-LADD (interaction 4)	2.1	0.99	0.03 *		
InAs ^k^ (cat. 0 = normal, 1 = high)				0.09	0.04 *
GSTP1 ^j^ (AG + GG vs. AA)	0.74	0.38	0.05		
GSTT1 ^l^ (Active vs. null)	0.54	0.40	0.17		
As3MT ^c^ (TG + GG vs. TT)	−1.6	1.1	0.14		
LADD ^g^ (cat. 0 = low, 1 = high)	−0.54	0.41	0.19		
GSTP1-GSTT1 (interaction 1)	1.5	1.2	0.21		
As3MT-LADD (interaction 2)	−0.93	0.54	0.08		

Note: * likelihood-ratio test logistic regression (*p* < 0.05). ^a^ SE = Standard error. ^b^ Monomethylarsonic acid dichotomized. ^c^ Dominant model for As3MT. ^d^ Basic model for *GSTM1*. ^e^ Age, dichotomized. ^f^ Body mass index, dichotomized. ^g^ Lifetime average daily dose exposure to arsenic (LADD), dichotomized. ^h^ Dimethylarsinic acid, dichotomized. ^i^ Dominant model for *GSTO2*. ^j^ Dominant model for *GSTP1*. ^k^ Inorganic arsenic dichotomized. **^l^** Basic model for *GSTT1*.

## Supplementary Materials

Supplementary materials can be found at https://www.mdpi.com/1422-0067/21/14/4832/s1.
