# Supplementary material for: As3MT and GST Polymorphisms Influencing Arsenic Metabolism in Human Exposure to Drinking Groundwater
Source: Int J Mol Sci. 2020 Jul 8;21(14):4832. doi: 10.3390/ijms21144832 (PMC7402318; doi:10.3390/ijms21144832)
Supplement: Supplementary file 1 [file ijms-21-04832-s001.pdf]

Supplementary Material

As3MT and *GST* polymorphisms influencing arsenic metabolism in human exposure to drinking groundwater

Farith González- Martínez[1,4], Daniel Sánchez-Rodas[2], Nelson M. Varela[3,4], Christopher A. Sandoval[4], Luis A. Quiñones[3,4], Boris Johnson-Restrepo[1]

[1] Environmental Chemistry Research Group and Public Health Research Group, University of Cartagena,130015 Cartagena, Colombia.
[2] Center for Research in Sustainable Chemistry, CIQSO. University of Huelva, Huelva 21071, Spain.
[3] Laboratory of Chemical Carcinogenesis and Pharmacogenetics (CQF). Department of Basic-Clinical Oncology (DOBC), Faculty of Medicine, University of Chile, Santiago, Chile.
[4] Latin American Network for Implementation and Validation of Clinical Pharmacogenomics Guidelines (RELIVAF-CYTED).

*Corresponding Authors:

Boris Johnson-Restrepo
Environmental Chemistry Research Group, School of Exact and Natural Sciences, University Campus of San Pablo, University of Cartagena. Zaragocilla. Carrera 50 No. 24-99. Cartagena 130015, Colombia. E-mail: bjohnsonr@unicartagena.edu.co Tel: +57-301-764-0553

Luis A. Quiñones
Laboratory of Chemical Carcinogenesis and Pharmacogenetics (CQF), Department of Basic-Clinical Oncology, Faculty of Medicine, University of Chile, Santiago, Chile. E-mail: lquinone@med.uchile.cl Tel. +56-2-29770741 (44)

Contents……………………………………………………………………………………….Page

Table S1. Primers and TaqMan probes used for genotyping of GSTs and As3MT with direct PCR and Real-Time PCR ……………………………………………………………………………3

Table S2. PCR conditions for genotyping.……………………………………………………….….3

Table S3. Physico-chemical parameters in water samples of study population……….………...4

Table S4. Lifetime average daily dose exposure to arsenic in drinking water according to arsenic concentrations
in groundwater above and below the optimal values……………………..……………………5

Table S5. Genotype and allele frequencies (%) for individuals from study population and report genotype frequencies from other studies………………………………………………………..6

Table S6. Effect of Lifetime average daily dose exposure to arsenic on urinary arsenic species, adjusted by covariates……………………………………………….……………………..7

Table S7. Urinary arsenic species concentration by polymorphic variants ………………..…….8

Table S8. Urinary arsenic species concentration by covariates…….. ……..………………………..9

Figure S1. Locations of the eight villages of study in the municipalities of Margarita and San Fernando, Colombia (Map from Google Earth® software)…………………………………...10

Figure S2. Comparison between arsenic concentrations in groundwater well at two different time points in study population. The medians were compared through Wilcoxon rank sum test…………………………………………………………………………………...………11

Table S1. Primers and TaqMan probes used for genotyping of GSTs and As3MT with direct PCR and Real-Time PCR.

Gene name	Symbol	Gene ID	Primers<br>Forward (5'→3')	Primers<br>Reverse (5'→3')	Size bp
Glutathione-s- tranferase T1	GSTT1	-	TTCCTTACTGGTCCTCACATCTC	TCACCGGACATGGCCAGCA	549
Beta Globin (Housekeeping gene)	*ß-globin*	-	CAACTTCATCCACGTTCACC	GAAGAGCCAAGGACAGGTAC	268
Glutathione- s- tranferase M1	GSTM1	-	GAACTCCCTGAAAAGCTAAAGC	GTTGGGCTCAAATATACGGTGG	273
Cytochrome P450 1A1(Housekeeping gene)	CYP1A1	4646903	TAGGAGTCTTGTCTCATGCCT	CAGTGAAGAGGTGTAGCCGCT	340
Gene name	Symbol	Gene ID	Sequence detection primers used with TaqMan probes		
Glutathione- s- tranferase TO2	GSTO2	156697	CAACTCTACACCCTCTCTTACGTGA[**T/C**]TAGACTTCCGTCGGGACGCAGTCCT		
Glutathione- s- tranferase P1	GSTP1	1695	CGTGGAGGACCTCCGCTGCAAATAC[**A/G**]TCTCCCTCATCTACACCAACTATGT		
Arsenic-3-metil transferasa	As3-MT	3740400	GTGTCTCGAGACCTTTGTCCTCCCC[**G/T**]CACCCCTCGGCCCGCTGCCTGCCCT		

Table S2. PCR conditions for genotyping.

Gene	Initial denaturation step	Denaturation	Annealing	Polymerization	Final polymerization	Cycles
GSTT1[a]	95 ºC x 7 m	95 ºC x 60 s	64 ºC x 60 s	72 ºC x 60 s	72 ºC x 5 m	32
GSTM1[b]	95 ºC x 5 m	95 ºC x 45 s	64 ºC x 45 s	72 ºC x 45 s	72 ºC x 5 m	32
GSTP1[c] rs1695	95 ºC x 10 m	95 ºC x 15 s	60 ºC x 90 s	60 ºC x 90 s		50
GSTTO2[d] rs156697	95 ºC x 10 m	95 ºC x 15 s	60 ºC x 60 s	60 ºC x 60 s		40
As3MT[e] rs3740400	95 ºC x 10 m	95 ºC x 15 s	60 ºC x 60 s	60 ºC x 60 s		40

[a]Caceres et al. 2005, direct PCR.
[b]Quiñones et al. 1999, direct PCR.
[c]Ramprasath et al. 2011, RTPCR.
[d]Rodrigues et al. 2012, RTPCR.
[e]Janasik et al. 2015, RTPCR

Table S3. Physico-chemical parameters in water samples of study population

Study área				Concentration As			Physico-chemical parameters of drinking water quality												
Location	Well	Latitude	Longitude	[a]As^{III}	[b]As^{V}	[c]TAs	[d]pH	[e]EC	[f]TDS	[g]T	[h]A	[i]TH	[j]DO	[k]Ca^{2+}	[l]Mg^{2+}	[m]Cl^{-}	[n]F^{-}	[o]NO_2^{-}	[p]NO_3^{-}
Mamoncito	1	9.022.668	74.469.300	24.8	10.5	35.3	7.15	570	302	10	358.4	251.7	2.70	66.5	20.8	12	4.6	0.01	1.09
Mamoncito	2	9.051.598	74.269.328	3.1	33.1	32.2	7.34	552	321	7.6	150.8	163.7	1.26	40.2	15.4	107.5	2.9	0.02	ND
Mamoncito	3	9.051.021	74.269.261	23.2	8.1	31.3	7.05	538	284	40	329.6	230.1	3.6	77.9	8.6	ND[q]	10.2	4.65	3.64
Mamoncito	4	9.050.937	74.269.144	25.6	8.1	33.7	7.37	642	315	6	284.7	230.5	5.43	66.8	15.5	ND	2.9	0.43	2.66
Mamoncito	5	9.551.112	74.268.881	23.2	10.5	33.7	6.90	487	257	45	291.3	241.3	2.1	64.1	19.7	10.3	0.47	3.65	2.34
Mamoncito	6	9.044.966	74.268.437	26.5	5.7	32.2	7.16	574	306	14	366.7	251.3	3.81	68.9	14.2	13.6	2.4	1.16	2.05
Mamoncito	7	9.050.015	74268103	5.5	26.6	32.1	6.92	654	346	70	436.7	278.9	2.81	71.6	23.3	14.2	10.2	4.15	5.32
Mamoncito	8	9.049.966	74.268.437	24.8	13.7	38.5	6.87	585	311	36	390.6	252.1	1.25	69.1	19.4	10.7	0.74	0.15	ND
Mamoncito	9	9.049.966	74.268.437	26.5	8.1	34.6	7.05	516	279	10	362.1	249.3	2.38	74.5	15.4	12.2	2.3	ND	1.07
Mamoncito	10	9.048.405	74.266.977	24.8	10.5	35.3	6.90	490	260	90	357.6	250.9	2.01	61.7	20.6	11.5	3.3	3.4	ND
Mamoncito	11	9.048.284	74.267.028	26.5	7.3	33.8	7.02	541	289	8.1	414.1	238.5	0.87	71.2	14.8	12.2	0.55	ND	1.05
Guataquita	12	9.028.805	74.216.208	24	18.5	42.5	6.71	398	210	50	241.0	196.9	1.44	64.1	8.9	19.5	3.8	2.88	1.88
Guataquita	13	9.020.556	74.215.236	23.2	4.1	27.3	6.71	384	204	37	251.3	206.1	0.50	63.3	11.7	21.6	2.5	1.22	2.11
La montaña	14	9.026.183	74.194.878	24	8.9	32.9	6.71	368	194	4.2	230.3	173.7	3.85	38.5	18.9	41.2	26.4	0.19	ND
La montaña	15	9.028.534	74.185.696	25.7	4	29.7	6.84	483	256	55	335.0	174.9	2.61	49.2	12.6	13.2	11.6	0.41	ND
Causado	16	9.132.035	74.319.918	3.1	36.3	39.4	7.09	681	361	7.7	443.3	236.9	2.54	56.6	23.2	31.6	7.0	4.8	5.3
Causado	17	9.129.371	74.319.275	23.2	20.2	43.4	7.12	669	354	10	251.6	234.5	3.38	53.5	24.5	20.3	6.8	4.6	5.25
Botón D.	18	9.152.282	74.253.617	23.2	43.5	66.7	7.48	144	74	120	59.3	102.0	5.78	30.9	6.03	16.3	4.7	0.17	ND
Santa rosa	19	9.088.956	74.312.169	23.2	12.9	36.1	6.62	374	192	1.8	250.5	158.1	1.9	32.1	18.9	10.9	3.7	0.01	ND
Santa rosa	20	9.088.411	74.311.338	24.8	1.6	26.4	6.85	553	298	95	207.6	260.1	1.28	45.2	35.8	12.4	7.9	0.52	ND
El porvenir	21	9.126.262	74.319.019	23.2	1.6	24.8	7.05	516	279	45	420.7	232.9	2.38	59.7	14.3	13.8	0.22	4.15	2.41
Margarita	22	9.090.110	74.170.170	6.0	4.0	10.0	6.85	510	270	30	300.5	220.5	2.55	57.5	16.3	19.1	0.35	1.80	1.75
Median				23.6	9.7	33.3	6.98	503.9	268.9	36.3	306.3	219.7	2.57	58.4	17.3	20.1	3.5	1.74	1.74
WHO Guideline value						10	6.5-8.5	400	500	5	-	300	6	100	150	250	1.5	3	50

Note: [a]As^{III} = arsenite (µg/L). [b]As^{V} =arsenate (µg/L). [c]TAs= Total arsenic (µg/g creatinine). [d]pH= Hydrogen potential. [e]EC=Electrical conductivity (Ω/cm). [f]TDS=Total Dissolved Solids (mg/L). [g]T= Turbidity (NTU). [h]A=Alkalinity (mg/L). [i]TH=Total Hardness (mg/L). [j]DO=Dissolved Oxygen (mg/L). [k]Ca^{2+}=Calcium (mg/L). [l]Mg^{2+}=Magnesium (mg/L). [m]Cl^{-} = Chloride (mg/L). [n]F^{-}=Fluoride (mg/L). [o]NO_2^{-} =Nitrite (mg/L) (as NO_2^{-}). [p]NO_3^{-}= Nitrate (mg/L) (as NO_3^{-}). [q]ND=Not detected.

Table S4. Lifetime average daily dose exposure (LADD) to arsenic in drinking water according to arsenic concentrations in groundwater above and below the optimal values.

	High exposure to As	Low exposure to As	Total
Parameters LADD¤	Mean (Range)	Mean (Range)	Mean (Range)
C [a] (µg/L), median (range)	35.5 (17,66.7)	10 (10,10)	33.3 (10,66.7)
IR [b] (L/day)	2.5	2.5	2.5
ED [c] (years)	17.9 (14.2,20.6)	15.9 (13.2,18.6)	17.2 (13.2,20.6)
EF [d] (days/year)	365	365	365
BW [e] (kg)	64.2 (36.9,98)	65.3 (48.7,84)	64.6 (36.9,98)
AT [f] (day/year)	27375	27375	27375
Total [g] (µg/kg-bw/day), median (range)	0.33 (0.14,0.65)	0.08 (0.06,0.11)	0.30 (0.06,0.65)
HQ [h] (ratio), median (range)	1.1 (0.43,2.1)	0.29 (0.22,0.39)	0.99 (0.22,2.1)

Note: ¤ LADD=Lifetime average daily dose exposure of As.
[a]C= concentration of arsenic in water.
[b]IR= water intake rate.
[c]ED= exposure duration.
[d]EF= exposure frequency.
[e]BW= body weight.
[f]AT= average time (lifetime expectancy of 27,375 day =75 years x 365 day).
[g]Total= ecuation 1.
[h]HQ (Hazard Quotient) = LADD/RfD= Reference Dose Arsenic (0.3 µg/kg bw/day).

Table S5. Genotype and allele frequencies (%) for individuals from study population and report genotype frequencies from other studies.

Countries/sample	Genotype frequency %		Genotype and Allele frequency %					
	GSTT1	GSTM1	GSTP1-rs1695		GSTO2-rs156697		AS3MT-rs3740400	
	*1/*0[a]	*1/*0[b]	AA/AG/GG	AG	TT/TC/CC	TC	TT/TG/GG	TG
**Colombia**								
Present study n=151	78/22	69/31	45/42/13	66/34	68/27/5	81/19	41/41/18	62/38
González et al. 2018 n=101	83/17	70/30	39/46/15					
**Chile**								
Cáceres et al. 2010 n=62	87/13	52/48						
Marcos et al. 2006 n=207	85/15	58/42	42/45/13	64/36				
**México**								
Recio et al. 2016 n=332	72/28	87/13						
**Argentina**								
Engstrom et al. 2007 n=112	83/17	39/61						
Engstrom et al. 2011 n=169							55/36/9	73/27
**Spain**								
García et al. 2005 n=1121	78/22	50/50						
**Italy**								
Boccia et al. 2007 n=553	84/16	47/53						
**Poland**								
Janasik et al. 2015 n=149	NA	NA	35/55/10	63/37	45/47/8	68/32	34/45/21	57/43
**Japan**								
Tamaki et al. 2011 n=203	49/51	50/50						
**China**								
Zhong et al. 2006 n=196		44/56	65/31/4	78/22				
Fu et al. 2013 n=66					47/44/9	69/31		
**Taiwan**								
Chung et al. 2011 n=251					53/41/6	74/26		
**Vietnam**								
Agusa et al. 2010 n=100					61/34/5	78/22		
Agusa et al. 2012 n=190			68/32/0	84/16				
**India**								
Ramprasath et al. 2011 n=270	82/18	79/21	44/48/8	68/32				

Table S6. Effect of Lifetime average daily dose exposure to arsenic on urinary arsenic species, adjusted by covariates

Urinary As Species	median (IQR)[a]	*p*-value raw	*p*-adjusted age	*p*-adjusted BMI[b]	Effect size Cohen´s
InAs[c]	0.80 (0.5,1.3)	0.97	0.86	0.81	0.21
MMA[d]	0.60 (0.30,1.3)	0.08	0.04*	0.05	0.52
DMA[e]	1.7 (1.1,2.8)	0.03*	0.02*	0.05	0.53
TuAs[f]	4.0 (2.7,7.0)	0.07	0.02*	0.05	0.48
N	151 (100)				

Note: *statistical significance t-test $p<0.05$, corrected by Bonferroni test.

[a] IQR= interquartile range defined as the range from the 25th percentile to the 75th percentile.

[b]BMI=body mass index.

[c]InAs= inorganic arsenic (As^{3+} and As^{5+}).

[d]MMA=Monomethylarsonic acid.

[e]DMA =Dimethylarsinic acid.

[f]TuAs= total urinary arsenic (µg/g creatinine-adjusted).

Table S7. Urinary arsenic species concentration by polymorphic variants.

		Total Arsenic median (IQR)[a]	Urinary arsenic species median (IQR)		
Genotypes ¤	N (%)	TuAs[b]-µg/g	InAs[c]-µg/L	MMA[d]-µg/L	DMA[e]-µg/L
All	151 (100)	4.0 (2.7,7.0)	0.80 (0.5,1.3)	0.60 (0.3,1.3)	1.7 (1.1,2.8)
GSTM1					
Active (AG or GG)	104 (68.9)	3.6 (1.3,5.7)	0.70 (0.40,1.1)	0.60 (0.30,0.98)	1.5 (1.0,2.5)
Null (none)	47 (31.1)	4.3 (2.9,7.9)	0.90 (0.50,1.6)	0.80 (0.40,1.6)	2.1 (1.6,5.4)
p-value		0.10	0.15	0.02*	0.02*
GSTT1					
Active (AG or GG)	118 (78.2)	3.7 (2.4,6.2)	0.70 (0.40,1.1)	0.6 (0.3,1.1)	1.7 (1.2,2.8)
Null (none)	33 (21.9)	4.7 (3.4,10.4)	1.0 (0.60,1.6)	0.7 (0.3,1.9)	1.7 (1.3,5.6)
p-value		0.30	0.11	0.37	0.92
GSTP1-rs1695					
AA (wild-type)	63 (41.7)	4.2 (2.9,6.9)	0.70 (0.40,1.2)	0.60 (0.3,1.2)	2.0 (1.4,3.0)
AG + GG	88 (58.3)	3.6 (2.2,6.7)	0.80 (0.50,1.3)	0.60 (0.3,1.2)	1.4 (0.9,2.4)
p-value		0.22	0.57	0.75	0.04*
GSTO2-rs156697					
TT (wild-type)	102 (67.5)	4.0 (2.7,6.9)	0.70 (0.4,1.1)	0.6 (0.3,2.1)	1.7 (1.2,2.8)
TC + CC	49 (32.5)	4.1 (2.7,5.1)	0.80 (0.4,1.2)	0.7 (0.3,2.2)	1.6 (1.0,2.8)
p value		0.81	0.26	0.36	0.64
As3MT-rs3740400					
TT (wild-type)	62 (41.1)	3.6 (1.4,4.3)	0.75 (0.35,1.1)	0.50 (0.10,0.83)	1.7 (1.2,2.9)
TG + GG	89 (58.9)	4.3 (3.0,8.7)	0.80 (0.50,1.3)	0.70 (0.40,1.5)	1.8 (1.2,3.1)
p-value		0.02*	0.27	0.04*	0.39

Note: *statistical significance Wilcoxon rank sum test was used to compare the urinary arsenic metabolites by genotype ($p < 0.05$), corrected by Bonferroni test. ¤HWE by chi-square test ($p > 0.05$).

[a]IQR= Interquartile range defined as the range from the 25th percentile to the 75th percentile.

[b]TuAs= total urinary arsenic creatinine-adjusted.

[c]InAs = inorganic arsenic (As^{III} and As^{V}).

[d]MMA= monomethylarsonic acid.

[e]DMA= dimethylarsinic acid.

Table S8. Urinary arsenic species concentration by covariates

		Total Arsenic median (IQR)[a]	Urinary arsenic species median (IQR)		
Covariates	N (%)	TuAs[b]-µg/g	InAs[c]-µg/L	MMA[d]-µg/L	DMA[e]-µg/L
All	151 (100)	4.0 (2.7,7.0)	0.80 (0.5,1.3)	0.60 (0.3,1.3)	1.7 (1.1,2.8)
Sex					
Female	43 (28.5)	4.2 (2.8,9.3)	0.80 (0.40,1.6)	0.7 (0.30,1.4)	2.0 (1.5,3.1)
Male	108 (71.5)	3.6 (2.3,6.6)	0.80 (0.50,1.2)	0.6 (0.30,1.2)	1.5 (0.9,2.6)
p-value		0.19	0.31	0.04*	0.21
BMI[f] (kg/m^2)					
Normal range	72 (47.7)	4.1 (2.7,7.1)	0.80 (0.30,1.2)	0.65 (0.25,1.3)	1.65 (1.0,2.5)
Underweight	79 (52.3)	3.9 (2.6,6.3)	0.70 (0.40,1.2)	0.60 (0.3,1.4)	1.90 (1.3,3.1)
p-value		0.69	0.80	0.59	0.45
Smoking Habit					
No	136 (90.1)	4.0 (2.7,7.0)	0.80 (0.5,1.3)	0.60 (0.3,1.3)	1.7 (1.1,2.8)
Yes	15 (9.9)	5.0 (3.6,7.1)	0.80 (0.4,1.1)	0.70 (0.4,3.0)	1.9 (1.3,5.1)
p-value		0.17	0.72	0.31	0.31
Alcohol consumption					
No consumption	114 (75.5)	4.2 (2.9,7.6)	0.80 (0.5,2.4)	0.70 (0.40,1.4)	1.8 (1.2,2.8)
≥5 glass weekend	37 (24.5)	3.3 (1.9,5.4)	0.60 (0.2,0.65)	0.50 (0.10,0.85)	1.4 (0.7,2.2)
p-value		0.04*	0.03*	0.07	0.17
Shellfish and/or fish					
< 2x per week	135 (89.4)	4.1 (2.8,6.9)	0.80 (0.50,1.3)	0.60 (0.3,1.3)	1.7 (1.1,2.8)
≥2x per week	16 (10.6)	3.1 (0.9,8.8)	0.55 (0.15,1.6)	0.55 (0.15,1.1)	1.4 (0.7,2.5)
p-value		0.30	0.34	0.74	0.39

Note: *statistical significance Wilcoxon rank sum test was used to compare the urinary arsenic metabolites by covariates ($p < 0.05$), corrected by Bonferroni test.
[a]IQR= Interquartile range defined as the range from the 25th percentile to the 75th percentile.
[b]TuAs= total urinary arsenic creatinine-adjusted.
[c]InAs = inorganic arsenic (As^{III} and As^{V}).
[d]MMA = monomethylarsonic acid.
[e]DMA = dimethylarsinic acid.
[f]BMI= body mass index.

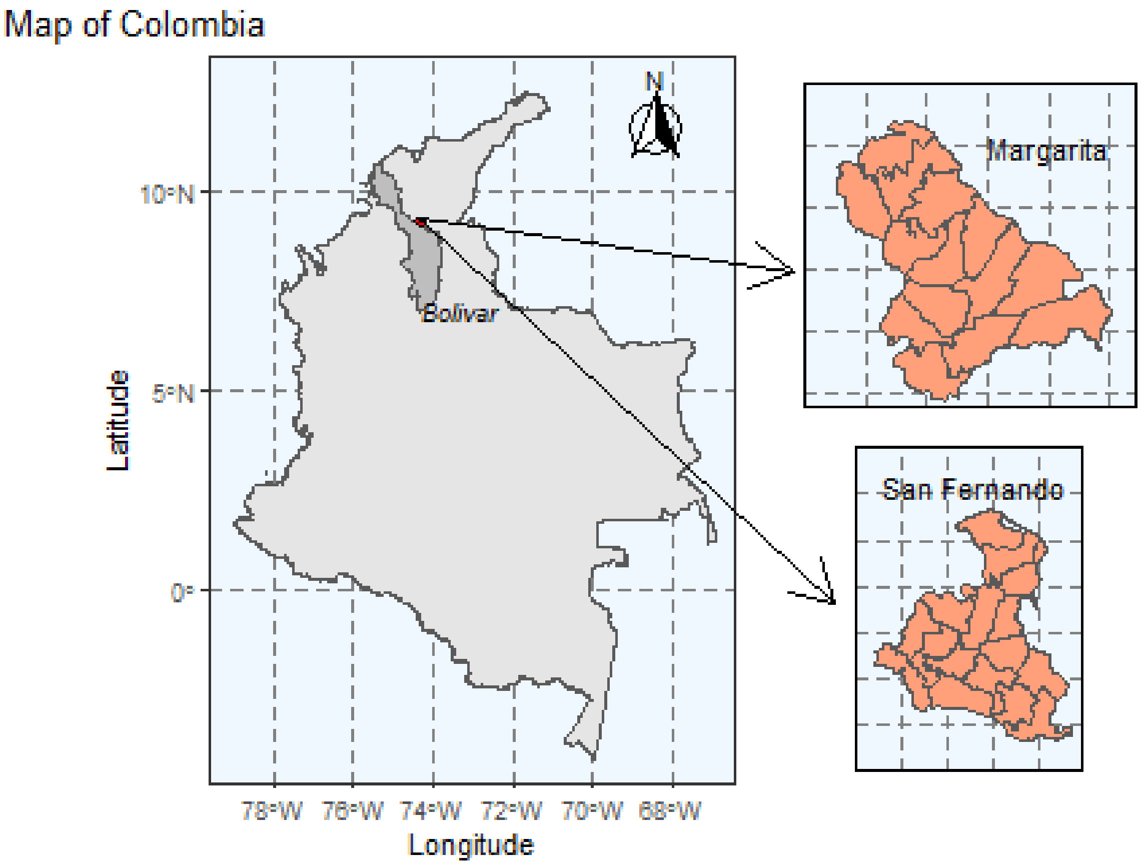

Figure S1. Locations of the eight villages of study in the municipalities of Margarita and San Fernando, Colombia

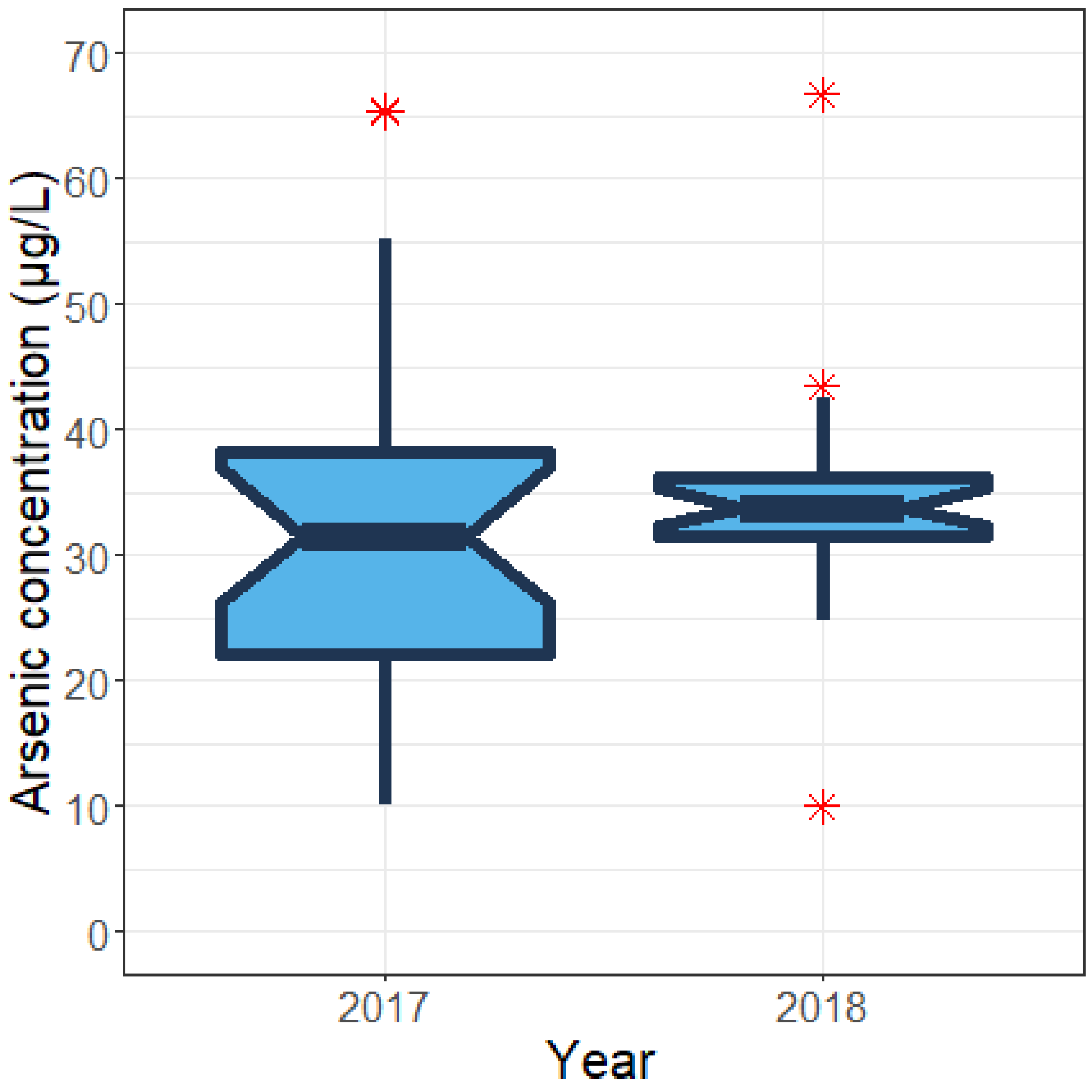

Figure S2. Comparison between arsenic concentrations in groundwater well at two different time points in study population. The medians were compared through Wilcoxon rank sum test (*p*>0.05).
